# Partitioning for Enhanced Statistical Power and Noise Reduction: Comparing One-Way and Repeated Measures Analysis of Variance (ANOVA)

**DOI:** 10.7759/cureus.75322

**Published:** 2024-12-08

**Authors:** Frederick Strale

**Affiliations:** 1 Biostatistics, The Oxford Center, Brighton, USA

**Keywords:** clinical trials, experimental trials, f-statistic, one way analysis of variance (anova), partitioning sums of squares, repeated measures analysis of variance (anova), repeated-measures design, statistical power

## Abstract

Using simulated data with duplicate observational data points, this research aims to highlight the notable efficiency of repeated measures analysis of variance (ANOVA) compared to one-way ANOVA as a more powerful statistical model. One of the principal advantages of repeated measures ANOVA is its design, in which each subject acts as their own control. This methodology allows for the statistical mitigation of individual differences among subjects, thereby reducing extraneous variability (noise) that can obscure the effects of the experimental conditions under investigation. By employing identical simulated column values within this analysis, we observe that the F-statistic generated by the repeated measures ANOVA tends to be larger than that derived from the one-way ANOVA.

A distinguishing feature of repeated measures ANOVA is its incorporation of an additional dimension of within-subject variation in its partitioning procedure. This acknowledges that measurements taken from the same subject are inherently correlated. This correlation introduces a separate source of partitioned variation, distinct from that attributable to between-subject differences. The term SS_Between x Within_ encapsulates the residual variation that remains after accounting for both group differences and individual subject discrepancies.

By explicitly recognizing the interrelatedness of measurements collected from the same subjects, repeated measures ANOVA effectively reduces the residual error variation contributing to the denominator in calculating the F-statistic. This reduction in error variation (noise) results in a more sensitive statistical test than one-way ANOVA, thus enhancing the power of the analysis. Consequently, the ability of repeated measures ANOVA to account for the correlated nature of repeated observations not only yields a more robust estimation of the treatment effects but also fortifies the statistical conclusions drawn from the data.

## Introduction

In statistical modeling, two of the many types of analyses of variance (ANOVA) are the one-way ANOVA and the repeated measures ANOVA. The one-way ANOVA (an extension of the two independent sample t-tests) is used to determine statistically significant differences between the means of three or more independent groups [1]. Repeated Measures ANOVA (an extension of the paired t-test) is used to analyze one group of subjects measured over three or more measurement instances. This usually occurs when participants are measured multiple times to investigate the impacts of an intervention longitudinally or when participants are subjected to more than one condition/trial and the response to each of these conditions wants to be compared [2-4,5].

The repeated measures ANOVA is considered more powerful than the one-way ANOVA because it accounts for the individual differences of subjects over time or conditions in partitioning procedures, which effectively reduces error variation (noise). The subjects serve as their own controls. In an experimental context, longitudinal measurements are taken from the same subjects at multiple time points, such as Baseline, Time 2, and Time 3. This design may allow for the direct attribution of any observed differences across these time points to the experimental treatment, subject to the limitations of carryover and order effects. By controlling for individual variability, this approach may ensure that the changes are not confounded by between-group differences (as is likely in one-way ANOVA), thereby isolating the effect of the treatment itself. The statistical reasoning behind this increased power stems from how it partitions the variation and deals with correlations in the data [5,6].

In a one-way ANOVA, the total variation in the data, namely, the total sums of squares (SST) is partitioned into two components. Between-groups sums of squares (SSB) is the variation due to differences between the group means, analogous to the mean difference in the two independent sample t-tests. It reflects how much the groups differ from each other. Within-groups sums of squares (SSW) represent the variation within each group due to individual differences or random error, analogous to the Standard Error of the Difference in the two independent sample t-test and paired t-test [5,7].

For one-way ANOVA, the test statistic F is computed as the ratio of the two mean squares (MS), where F=MS_Between_ (MSB)/MS_Within_ (MSW), where MS_Between_ is the mean square for between-group variation, MS_Within_ is the MS for within-group (error) variation. The one-way ANOVA treats each subject as an independent observation, which leads to higher error variability, especially when individual differences, inherent in 2+ group designs are present but ignored [5,7].

The repeated measures ANOVA has an additional component of within-subjects variation which takes into account that measurements from the same subject are related. This design partitions the total variation into SS_Between_, as in one-way ANOVA, where this reflects the variation due to differences between the group means across conditions or time points. SS_Within_, the variation within subjects across time or conditions, represents individual differences (how much subjects vary from each other). This is the variation between the individual subjects, and it is treated as a separate source of variation [5,6].

SS_Between x Within_ represents the partitioned residual variation after accounting for both group differences and subject differences. By accounting for the correlated nature of within-subject measurements, this residual error variation is what contributes to the smaller denominator in the F-statistic calculation as compared with one-way ANOVA F [5,6].

For repeated measures, ANOVA, the F is computed as F = MS_Between_ divided by MS_Between x Within_ where "MS_Between x Within_" represents the mean square for residual error variation after accounting for subject-specific effects [5,6].

The key difference in repeated measures ANOVA is that individual variability is partitioned separately, which reduces the error term (MS_Between x Within_). By removing this within-subject variation from the error term, the denominator of the F-statistic is smaller, making the F-ratio larger and increasing statistical power [5,6].

Given these explanations, this study aims to compare the statistical power and sensitivity of one-way ANOVA and repeated measures ANOVA using identical simulated datasets. By partitioning the sums of squares, this study will demonstrate that repeated measures ANOVA explicitly accounts for the interrelatedness of measurements collected from the same subjects. This approach effectively reduces the residual error variation in the denominator of the F-statistic calculation, resulting in a larger F-statistic. Consequently, this reduction in error variation (noise) leads to a more sensitive statistical test compared to one-way ANOVA, thereby enhancing the power of the analysis.

## Materials and methods

Study design: Data collection

This study compares two distinct statistical methodologies, one-way ANOVA and repeated measures ANOVA, by evaluating their performance using simulated datasets derived from identical observational data points. The data are generated from two experimental conditions, one representing a cross-sectional design, analyzed using one-way ANOVA, and the other representing a longitudinal design, evaluated using repeated measures ANOVA.

Both statistical approaches are applied to datasets involving identical numbers, three distinct groups, and three separate time points to investigate potential differences in group means across these conditions. Through this comparative analysis, the present study seeks to elucidate the statistical power advantages inherent in repeated measures ANOVA, characterized by a larger F-statistic, with identical observational data points, as opposed to one-way ANOVA. This investigation aims to delineate the appropriateness of each methodological approach in analyzing group mean differences within experimental designs of varying structures. The study endeavors to determine the efficacy and statistical significance of treatment effects by providing a robust framework for selecting the most suitable analytical method in experimental research.

Data simulation: One-way ANOVA

This dataset, analyzed using a one-way ANOVA, enables a comparative examination of three distinct groups: the Control Group, Experimental Group #1, and Experimental Group #2. The data for each group are presented in Table 1 below.

**Table 1:** One-Way ANOVA M: mean; n: #of observations; s: standard deviation

Control Group	Experimental Group #1	Experimental Group #2
53	47	45
49	42	41
47	39	38
42	37	36
51	42	35
34	33	33
44	13	46
48	16	40
35	16	29
18	10	21
32	11	39
27	6	20
M=40	M=26	M=34.4
n=12	n=12	n=12
s=10.796	s=15.202	s=8.394

These values above represent the outcomes for 12 independent observations from each group. The groups are assumed to be independent, with no repeated measurements across groups [5].

Data simulation: Repeated measures ANOVA

The repeated measures ANOVA analyzes data collected from the same subjects at three different timepoints. The data below have identical sample sizes, datapoints, and means as with the previous one-way ANOVA scenario. Please see Table 2 below. The data represent measurements taken from 12 subjects at three timepoints: Time 1 (Baseline), Time 2, and Time 3. The measurements at each timepoint for each subject are treated as repeated measures [5].

**Table 2:** Repeated Measures ANOVA -=Empty cell; M: mean; n=#of observations; s: standard deviation

Subject #	Baseline	Time 1	Time 2
1	53	47	45
2	49	42	41
3	47	39	38
4	42	37	36
5	51	42	35
6	34	33	33
7	44	13	46
8	48	16	40
9	35	16	29
10	18	10	21
11	32	11	39
12	27	6	20
-	M=40	M=26	M=34.4
-	n=12	n=12	n=12
-	s=10.796	s=15.202	s=8.394

Statistical analyses: One-Way ANOVA

A one-way ANOVA was conducted to determine significant differences in the mean values between the three independent groups: Control, Experimental Group #1, and Experimental Group #2. The model for this analysis is expressed as:

Y_ij_ =μ+τ_i_ +ϵ_ij _

where Y_ij_ represents the observed value for the jjj-th subject in the iii-th group, μ (mu) is the overall mean, τ (tau) is the effect of the iii-th group, and ϵ (epsilon)is the random error associated with the jjj-th subject in the iii-th group.

Statistical analyses: Repeated measures ANOVA

To evaluate the impact of time on the outcome across the three timepoints, a repeated measures ANOVA was performed. This analysis accounts for the correlation between measurements from the same subjects at different timepoints. The model for this analysis is expressed as:

​Y_ij_k =μ+α_i _+β_j_ +(αβ)_ij_ +ϵ_ij_k

where Y_ij_k is the observed value for the kkk-th measurement of the iii-th subject at the jjj-th timepoint, μ (mu) is the overall mean, α (alpha) represents the subject-specific effect, β (beta) is the effect of the timepoint, and (αβ) (alpha beta) is the interaction between the subject and timepoint. The ϵ (epsilon) term represents the residual error.

Statistical analysis summary

All statistical analyses for the one-way ANOVA and repeated measures ANOVA were performed using the Statistical Package for the Social Sciences (SPSS), version 29.0. With the one-way ANOVA, this technique was employed to assess the mean differences between independent groups across a single factor or condition with three levels (groups). One-way ANOVA is particularly useful in experimental designs where multiple groups are compared at a single point in time. The primary objective of this analysis was to determine whether there were statistically significant differences in group means.

The repeated measures ANOVA was utilized to evaluate the differences in group means over multiple time points (three in this study), specifically in the context of longitudinal data. This analysis accounts for the correlated nature of the repeated observations within the same subjects, allowing for the detection of within-subject changes over time while adjusting for the dependency between measurements.

Both statistical methods were applied to simulated datasets. Identical observational datapoints, comprising three distinct groups, or measured at three distinct time points were used. This enabled a direct comparison of the efficacy of each method in detecting group mean differences across cross-sectional (One-way ANOVA) and longitudinal (Repeated measures ANOVA) experimental designs.

Institutional Review Board (IRB): Human subjects

This research involves the use of simulated data that does not involve any real human participants or the collection of identifiable private information. As such, it does not fall within the regulatory definition of human subjects’ research as outlined by the U.S. Department of Health and Human Services (HHS) and the U.S. Food and Drug Administration (FDA). The study is designed exclusively for educational purposes, including but not limited to, classroom exercises, training simulations, and methodological explorations aimed at illustrating statistical concepts and model applications.

In accordance with federal regulations (45 CFR 46 and 21 CFR 50), research that exclusively involves the use of simulated or synthetic data and does not include human participants or the use of identifiable personal information, is typically exempt from IRB review. This exemption applies because the research does not engage with any actual human subjects, nor does it involve any activities that would pose a risk to individuals' privacy or confidentiality.

Because the study’s objectives are confined to exploring statistical methods, model performance, or other technical aspects of data analysis in a controlled, non-human context, it does not meet the threshold for requiring IRB oversight. Accordingly, no formal review by an IRB is required for the current study.

## Results

One-Way ANOVA

Table 3 below presents the data set and “ANOVA Table” for three independent groups. This table partitions the sources of variation into two primary components: between-groups variation and within-groups variation. The F-statistic, derived by dividing the MS between by the mean square within, yields a statistically significant value of 4.284, accompanied by a p-value of 0.022 [5].

**Table 3:** One-Way ANOVA Results -=Empty cell; M: mean, n=# of observations; s: standard deviation; Grand M: grand mean; Grand N: total number of subjects; SS: sums of squares; df: degrees of freedom; MS: mean square

Control Group	Experimental Group #1	Experimental Group #2			ANOVA Table			
53	47	45	Source of Variation	SS	df	MS	F	p-value
49	42	41	Between Groups	1194	2	597	4.284	0.022
47	39	38	Within Groups	4599	33	139.364	-	-
42	37	36	Total	5793	35	-	-	-
51	42	35	-	-	-	-	-	-
34	33	33	-	-	-	-	-	-
44	13	46	-	-	F=597/139.364=4.284	-	-	-
48	16	40	-	-	-	-	-	-
35	16	29	-	-	-	-	-	-
18	10	21	-	-	-	-	-	-
32	11	30	-	-	-	-	-	-
27	6	20	-	-	-	-	-	-
M=40	M=26	M=34.5	Grand M=33.5	-	-	-	-	-
n=12	n=12	n=12	Grand N=36	-	-	-	-	-
s=10.796	s=15.202	s=8.394	-	-	-	-	-	-

Repeated measures ANOVA

Table 4 below illustrates the dataset and “ANOVA Table” for a repeated measures ANOVA, encompassing three temporal measurements within a single cohort of research subjects. This table partitions the sources of variation into three distinct components: between-groups variation, within-groups variation, and the interaction term (between-groups × within-groups variation). The F-statistic, calculated by dividing the MS between by the MS of the interaction term, yields a statistically significant value of 12.305, with a p-value less than 0.001, thus suggesting a higher statistical power than one-way ANOVA [5]

**Table 4:** Repeated Measures ANOVA Results -=Empty cell; n=# of observations; Grand M: grand mean; Grand N=Total number of subjects; s: standard deviation; Grand M: grand mean; M: mean; SS: sums of squares; df: degrees of freedom; MS: mean square

Patient #	Time 1	Time 2	Time 3			ANOVA Table			
1	53	47	45	Source of Variation	SS	df	MS	F	p-value
2	49	42	41	Between Groups	1194	2	597	12.305	<0.001
3	47	39	38	Within Groups	3531.67	11	321.06	-	-
4	42	37	36	Between x Within	1067.33	22	48.52	-	-
5	51	42	35	Total	5793	35	-	-	-
6	34	33	33	-	-	-	-	-	-
7	44	13	46	-	-	-	-	-	-
8	48	16	40	-	-	F=597/48.52=12.305	-	-	-
9	35	16	29	-	-	-	-	-	-
10	18	10	21	-	-	-	-	-	-
11	32	11	30	-	-	-	-	-	-
12	27	6	20	-	-	-	-	-	-
-	M=40	M=26	M=34.5	Grand M=33.5	-	-	-	-	-
-	n=12	n=12	n=12	Grand N=36	-	-	-	-	-
-	s=10.796	s=15.202	s=8.394	-	-	-	-	-	-

## Discussion

The F-statistics for both one-way ANOVA (3 groups) and repeated measures ANOVA (3 points) have been reported as F = 4.284 and F = 12.305, respectively. Given that identical data were used for both analyses, it is essential to explore the implications of these F-statistic values for the statistical power of each design and the broader context of the experimental study [5].

The larger F-value for the repeated measures ANOVA may indicate greater statistical power because the error term (denominator of the F-statistic) is reduced by accounting for within-subject variation in its partitioning procedure. This may increase the likelihood of detecting a true effect. This also implies that the unexplained variation is lower, making it easier to detect differences due to experimental manipulation rather than random noise. With a smaller error term, the F-value becomes larger for the same amount of between-group variation [5,6-9].

A larger F-value in repeated measures ANOVA indicates a greater ratio of explained variation to unexplained variation, which may increase the sensitivity of the test to detect true effects. Given that statistical power is the probability of correctly rejecting the null hypothesis when it is false, reducing the error variance makes the test more powerful; namely, it has a higher chance of detecting a true effect if one exists. Therefore, by reducing the error term through accounting for within-subject variation, the repeated measures ANOVA may increase the likelihood of detecting a true effect as it enhances the signal-to-noise ratio, making true differences more apparent [5].

One-way ANOVA: Between-group differences

A one-way ANOVA tests for significant differences between three or more independent groups, in this case, three. The F-statistic (4.284) reflects the ratio of between-group and within-group variation. The higher the F-value, the greater the evidence against the null hypothesis that all group means are equal. In statistical power, an F-statistic of 4.284 suggests moderate evidence for rejecting the null hypothesis [1,2,7,9-11].

Statistical power, by definition, is the probability of correctly rejecting the null hypothesis when it is false. A moderate F-statistic, such as 4.284, typically indicates a large effect size (Eta^2^=0.206), corresponding to moderate power under typical sample sizes (e.g., n=20 per group). Although an F-statistic of 4.284, with a p-value equal to 0.022, is unlikely to be trivial, it might not fully exploit the potential for detecting differences, mainly if the sample size is relatively small or the effect size is moderate [1,2,7,8,12,13-16].

Therefore, the power to detect significant differences in the one-way ANOVA is likely to be moderate in this instance, and the analysis may require further adjustment, such as increasing the sample size or employing more sensitive post-hoc tests, to optimize the chances of uncovering true group differences [7,8].

Repeated measures ANOVA: Within-subjects design

In contrast, a repeated measures ANOVA evaluates differences within the same subjects across multiple time points, in this case, three. The F-statistic of 12.305 is notably larger than that observed for the one-way ANOVA (4.284). This discrepancy highlights an essential feature of repeated measures designs; they generally boost statistical power (Partial Eta^2^=0.528), which is a large effect by accounting for the correlation between repeated measurements within the same subjects. Because repeated measures design control for between-subject variability, the resulting error variance (noise) is often smaller, increasing the test’s power to detect meaningful within-subject effects [1-6,12,17-20].

The larger F-statistic of 12.305 may imply that the effect of the independent variable (e.g., time, intervention) is more pronounced in the context of the repeated measures, likely reflecting a larger effect size compared to the one-way ANOVA. This increased effect size, often represented by Eta^2^ or Partial Eta^2^, may suggest that repeated measures designs may more efficiently identify significant differences. In practical terms, the repeated measures design offers greater statistical power with the same data, reducing the likelihood of type II errors (failing to reject the null hypothesis when it is false) [1,2,5,6,8,10,11].

Comparative power implications

The one-way ANOVA lumps all within-subject variability into the error term, resulting in a smaller F-value. In the repeated measures ANOVA, the sums of squares are partitioned as follows.

SS_Total_ = total variation in the data. SS_Total_ = SS_Between_ + SS_Within_ + SS_Between x Within_. SS_Between_= due to between-group differences (like one-way ANOVA). SS_Within_ = captures the individual differences across subjects. SS_Between x Within_ represents the error that is not explained by either the between-group or subject-specific differences [5,7,9-11].

In the repeated measures design, the mean square for subjects (the MS_Within_) is not included in the calculation of the F-statistic because it represents a systematic source of variation (individual differences), not a random error. By isolating this variation, the error term, SS_Between x Within_, used in the F-statistic denominator, is smaller, resulting in a more powerful test [5,6,12].

The difference in F-statistics (4.284 for one-way ANOVA vs. 12.305 for repeated measures ANOVA) may underscore the enhanced statistical power of the repeated measures approach. The larger F-statistic in the repeated measures ANOVA may indicate that this design is more effective at detecting differences due to its inherent ability to control for within-subject variability. Since the same participants are used across all time points, the error variation (noise) in a repeated measures design is typically reduced, thereby increasing power relative to an independent groups design (as in one-way ANOVA) [5,11,13-20].

For identical sample sizes, the repeated measures ANOVA is more likely to detect true effects, suggesting that the analysis using repeated measures may be more efficient in terms of statistical power. This makes repeated measures an advantageous choice in situations where participants are available for multiple time points, as it maximizes the information gleaned from each participant, thus requiring fewer participants to achieve equivalent power compared to an independent group’s design [5-12,13-20].

Limitations

While this study provides valuable insights into the comparative statistical power of one-way ANOVA and repeated measures ANOVA, some limitations should be evaluated when interpreting the results. The use of simulated data, though beneficial for isolating specific statistical properties, inherently limits the generalizability of the findings to real-world experimental settings. Simulations often rely on predefined assumptions regarding the underlying data distribution, effect sizes, and homogeneity of variance, which may not fully capture the complexity and variability encountered in actual experimental data. As such, the conclusions drawn about the relative power of the two analyses should be viewed with caution when applied to diverse real-world datasets.

However, in practical scenarios, the likelihood of encountering real-world datasets where data points are perfectly identical is virtually nonexistent. Consequently, to facilitate a controlled comparison, this study employed a statistical data simulation. This simulation enabled the creation of identical datasets, ensuring the methodological equivalence necessary for a precise and unbiased comparison. Such an approach allowed for a direct, “apples-to-apples” evaluation, isolating the methodological differences in sums of squares partitioning, and their impact on their resulting F-statistics. By leveraging simulated datasets, this study provided a framework for examining the subtle distinctions and underlying mechanics of one-way ANOVA and repeated measures ANOVA. This approach not only standardized the datasets but also eliminated confounding variables that might have arisen in empirical data. Through this controlled environment, we assessed the particulars in F-statistic outcomes attributable to the structural, conceptual, and computational differences between these two ANOVAs.

The assumption of sphericity, an essential condition for repeated measures ANOVA, was not explicitly tested or addressed in this analysis. The failure to meet the assumption of sphericity can lead to inflated type I error rates, particularly when the degrees of freedom are adjusted using methods such as the Greenhouse-Geisser correction [12]. While this study’s methodology accounts for within-subject variability, it does not account for potential violations of this assumption, which could affect the validity of the results, especially in studies with multiple timepoints or conditions.

Nevertheless, this study is a methodologically straightforward simulation, with the standard assumptions underpinning one-way ANOVA and repeated measures ANOVA intentionally disregarded. This deliberate omission enabled the study to concentrate solely on the comparative analysis of F-statistics generated from identical datasets under these two distinct ANOVA paradigms. By isolating the computational processes from their associated assumptions, the analysis aimed to distill and highlight fundamental differences in the outcomes produced by these statistical techniques.

The impact of sample size was not systematically explored within the context of statistical power. While moderate F-statistics in the one-way ANOVA suggest the potential for detecting meaningful group differences, the power of the analysis is a function of factors such as sample size, effect size, and variation within groups [7,8]. A more robust investigation would involve examining the interaction between these factors to better understand how variations in sample size or effect size influence the power of both ANOVA models.

Additionally, the scope of this study was limited to a comparison of statistical power under conditions where the same data were analyzed using both methods. In practical applications, factors such as the time cost, participant burden, and experimental design constraints associated with repeated measures data collection may limit the feasibility of employing repeated measures ANOVA in some experimental contexts. The potential for carryover effects or order effects in repeated measures designs also warrants consideration, as these factors could introduce additional sources of error variance and bias the results [12].

## Conclusions

With the identical simulated datasets, while both one-way ANOVA and repeated measures ANOVA report significant findings (with F-statistics of 4.284 and 12.305, respectively), the latter demonstrates greater statistical power. This enhancement arises from the reduced error variance (noise) and the efficient use of within-subject variation in repeated measures designs. The larger F-statistic in the repeated measures context may suggest that this approach is better suited for detecting meaningful differences, mainly when the data consists of repeated observations from the same individuals. Therefore, researchers are encouraged to consider their study’s design carefully, as the choice between one-way ANOVA and repeated measures ANOVA has profound implications for the statistical power and sensitivity of the analysis.
